# Evaluation of a Smartphone-Based Weight Loss Intervention with Telephone Support for Merchant Women With Obesity in Côte d'Ivoire: Protocol for a Randomized Controlled Trial

**DOI:** 10.2196/69264

**Published:** 2025-03-18

**Authors:** Rui Usui, Maki Aomori, Shogo Kanamori, Setsuko Watabe, Bi Tra Jamal Sehi, Kei Kawano, Yuka Kanoya

**Affiliations:** 1 Department of Nursing Shonan University of Medical Science Yokohama, Kanagawa Japan; 2 Nursing Course School of Medicine Yokohama City University Yokohama, Kanagawa Japan; 3 Bureau of International Health Cooperation National Center for Global Health and Medicine Tokyo Japan; 4 Department of Human and Social Sciences Félix Houphouët-Boigny Univesity Abidjan Cote D'Ivoire

**Keywords:** West Africa, sub-Saharan Africa, obesity, noncommunicable diseases, mHealth, mobile health, eHealth, randomized controlled trial, Côte d'Ivoire, weight loss program

## Abstract

**Background:**

The obesity rate among women in Côte d'Ivoire is rising, particularly in urban areas. Merchantry is the leading occupation for women in the country, and merchant women face a high risk of obesity owing to their sedentary lifestyle. A previous survey indicated that the obesity rate among merchant women was 30%, double the national average. Furthermore, 82.2% of merchant women with obesity were unaware of their condition, and 40.1% expressed no interest in losing weight. While most weight loss programs target individuals ready to lose weight, community interventions should also address those with minimal readiness. Additionally, low-cost weight-loss interventions that do not require health professionals are needed in countries with limited medical resources. Smartphones could offer a cost-effective solution as they enable self-monitoring and remote communication.

**Objective:**

This study will evaluate a low-cost smartphone-based intervention that targets individuals who are not ready to lose weight without the involvement of health professionals.

**Methods:**

The intervention will run for 6 months, and its efficacy will be assessed in an unblinded, parallel-group, randomized controlled trial with 108 participants per group. All direct interventions for participants in this study will be carried out by staff without medical qualifications. The intervention group will receive weighing scales and be encouraged to record their weight with a smartphone app. Health education will be provided via weekly group messages and monthly phone calls. The evaluation will be conducted face-to-face. The primary outcome will be the weight change, and the secondary outcome will be differences in body fat percentage, abdominal circumference, and stage of behavioral change in weight loss behaviors from baseline to 3, 6, and 12 months.

**Results:**

In accordance with this protocol, the recruitment of participants started on August 26, 2024. A total of 216 participants were allocated, with 108 in the intervention group and 108 in the control group. The baseline survey began on November 15, 2024, and is currently ongoing as of the end of November 2024.

**Conclusions:**

This study will be the first in sub-Saharan African countries to implement a smartphone app-based weight loss program in sub-Saharan Africa that does not require direct intervention by health care professionals but specifically targets communities. Furthermore, if the effectiveness of this program is confirmed, it has the potential to serve as a low-cost sustainable weight loss model at the policy level.

**International Registered Report Identifier (IRRID):**

DERR1-10.2196/69264

## Introduction

### Background

Obesity caused an estimated 5 million deaths associated with noncommunicable diseases (NCDs) such as cardiovascular diseases, diabetes, cancers, and chronic respiratory diseases [1]. Additionally, its high prevalence in people 18 years and older is a critical issue, at 17.9% for women and 13.6% for men in 2022. In Africa, the obesity rate was 17% in women and 6.8% in men in 2022 [2]. This difference is particularly significant, making obesity among women an urgent issue. The percentage of women with obesity in Côte d'Ivoire has doubled compared to 20 years ago (7.3% in 2002 and 15.7% in 2022) [2].

The characteristics of the occupational style of African market traders indicate that these traders are at high risk of obesity due to long sedentary hours and easy access to food [3-5]. In Abidjan, the largest city in Côte d'Ivoire, 64.3% of working women are merchants [6], and merchant is a common occupation among women from lower- and middle-income groups. A survey of medical facilities in Abidjan also showed that merchantry was the most common occupation among patients with overweight and obesity [7]. Furthermore, our all-inclusive market survey showed that the obesity rate among merchant women is 30% [8], double the national average of 15.3%. This indicates an urgent need to address obesity among individuals in the merchant community.

Obesity is a substantial cause of NCDs such as heart disease and type 2 diabetes [9,10], and weight loss can reduce the risk of NCDs. However, in Côte d'Ivoire, there is no community-targeted weight loss program. In general, weight loss programs often target those who wish to lose weight. However, in African countries such as Côte d'Ivoire, where being plump is a symbol of wealth and power, many individuals with obesity have no desire to lose weight. Notably, 82.2% of merchant women with obesity who work at the targeted market were unaware that they had obesity, and 40.1% did not wish to lose weight (38.6% wished to maintain their current weight and 1.5% wished to gain weight) [8]. Thus, in an environment such as that in Côte d'Ivoire, developing measures to address obesity in communities that include those with minimal readiness for weight loss is necessary.

To increase the willingness of individuals with obesity to undergo weight loss, we must educate them on their condition and reasons to lose weight. Weighing is key to this; however, in our study, 53.3% of merchant women with obesity did not weigh themselves once a year, and 95.5% of them did not own a scale. Regular weighing is effective for weight loss even without interventions such as weight loss programs [11] and is one of the most cost-effective and easiest weight loss methods to implement. In this study, we aim to increase the awareness of one’s own weight through regular weighing, promote awareness of health risks, and increase readiness for weight loss.

Noninvasive exercise and diet are the cornerstones of behavioral therapy as a treatment method for obesity [12]; overweight and obesity management guidelines [13] that include behavior change programs have been established in the United States. However, these are primarily conducted by primary care physicians in medical institutions and require advanced medical personnel and resources such as frequent counseling and elaborate individualized behavioral strategy planning. Therefore, implementing these behavior change programs as is in resource-limited countries such as Côte d'Ivoire is challenging [14,15].

Mobile health is cost-effective and impactful in supporting diet and exercise regimens [16-20]. Self-monitoring using smartphones is more effective than other methods of weight management [20]. Enhanced social support through online social networking services (SNSs) can also be effective. For example, Facebook groups have shown weight loss benefits [21]. In Côte d'Ivoire, WhatsApp is a popular private communication tool; however, studies on implementing behavior change using WhatsApp are limited [22]. Most studies that use smartphone apps and SNSs have been conducted in high-income countries. Such studies are limited in sub-Saharan Africa because sociocultural backgrounds and the use of smartphones and SNSs are different.

The prevalence of smartphones in sub-Saharan Africa was 45% in 2019 and is expected to steadily increase [23]. In our previous study, 77% of female traders in the target markets used smartphones [8], suggesting that interventions using smartphones are feasible. Smartphone use is important in areas requiring low-cost interventions.

Community-based weight loss interventions in sub-Saharan Africa include a group session intervention in South Africa [24] and a program initiated in Burkina Faso that includes multiple in-person dietary counseling sessions with experts [25]. These include multiple in-person sessions with health professionals, counseling, and health education.

Considering their feasibility in areas where medical resources are scarce, smartphone use that allows for remote intervention and methods that require less intervention by health care workers are required.

In this study, we aim to examine the effectiveness of a low-cost versatile weight loss intervention that can be implemented without intervention by health professionals in a community that comprises those with minimal readiness for weight loss. Specifically, this study will examine the effectiveness of a weight loss intervention that incorporates weight measurement promotion, the use of a smartphone app, and communication with non–health professionals among female merchants with obesity in Côte d'Ivoire. This will be an unblinded, parallel-group, randomized controlled trial.

This paper outlines the study protocol of a randomized controlled trial, describing the intervention and examining its effectiveness.

### Hypothesis

This study hypothesizes that the intervention group will achieve a weight loss of at least 2% [13] from baseline to 6 months, significantly differing from the control group. We will consider 2% weight loss as a clinically meaningful value; since the average weight of women with obesity in the target market is 85.6 kg [8], the equivalent of 2% is –1.7 kg. Based on the results of previous studies [19], this goal was deemed feasible.

## Methods

### Research Design

This study will focus on market A in Abidjan, Côte d'Ivoire. The effectiveness of the weight loss intervention will be tested in a parallel-group (1:1) randomized controlled trial among Merchant women with obesity within this market. As standard treatment, the control group will be weighed and provided with their BMI based on physical measurements, whereas the intervention group will receive a scale and the weight loss intervention using a smartphone app, and the differences will be compared. The study will be unblinded, and the effectiveness of the program will be evaluated after 6 months. In addition, a follow-up evaluation will be conducted 12 months later to assess sustained changes after the intervention is complete. We followed the SPIRIT (Standard Protocol Items: Recommendations for Interventional Trials) 2013 statement and CONSORT-eHEALTH (Consolidated Standards of Reporting Trials of Electronic and Mobile Health Applications and Online Telehealth) checklist (V 1.6.1).

### Eligibility Criteria

The target population will be female merchants between 18 and 65 years of age working in market A with a BMI ≥30 who own a smartphone for personal use and who can use WhatsApp. The exclusion criteria included those who are pregnant or lactating, those who are found pregnant during the intervention period, those for whom weight loss is contraindicated (due to a cancer diagnosis, eating disorders, poorly controlled hypertension, diabetes, etc), and those using physician-prescribed weight loss medication. If they have medical records, we will review them for the eligibility assessment. If they do not, we will ask about their conditions in detail and make a judgment.

### Recruitment

Four interviewers and supporters will visit the target market, approach potential participants, and screen them to see if they satisfy the inclusion criteria by asking a few questions and taking physical measurements (weight and height measurements). If the participants were eligible, we explained the purpose of the study, the intervention and procedures, and the eligibility and exclusion criteria in detail and returned at least half a day later to obtain their signatures on a consent form. We also explained that the participants would be randomly assigned to either the intervention or control group and would not be able to self-select. We explained that all participants would be weighed regularly at the beginning of the intervention as well as 3, 6, and 12 months later, and that their height would be measured at the beginning of the study so that their BMI would be known each time. We further explained that the control group will have access to free scales placed at several locations in market A 12 months after the end of the entire study, which they can use at their leisure. We also informed them that smartphone apps and other devices would be introduced to them at the end of the study.

Participants could withdraw from the study at any time before data collection at 6 months by submitting a consent withdrawal form to a member of the research team.

### Intervention

The weight loss intervention implemented in this study will target communities, including those who have no desire to lose weight, and will be low-cost without direct intervention by health professionals. The basic framework will be based on the American Heart Association (AHA)/American College of Cardiology (ACC)/The Obesity Society (TOS) guidelines for the management of overweight and obesity in adults [13] and the Burkina Faso program [25], which is implemented in the sociocultural context of West Africa. However, since both of these programs include direct intervention by health professionals, which differs from the purpose of this study, the content of the intervention is adapted to the situation in Abidjan in this study (Table 1). The weight loss intervention will be conducted for 6 months. The intervention will be based on self-weighing [11], which has been shown to be beneficial for weight loss, and will include the use of a low-cost personalized support smartphone app [16]. In addition, direct contact with the intervention group will be conducted by Ivorian non–medical professional supporters who were selected based on their research experience within Côte d'Ivoire and have received training for this study.

The intervention consisted of the following three components: (1) promoting weight measurement and encouraging self-monitoring through a smartphone app; (2) health education using a messaging app; and (3) in-person or telephone health education based on the stage of behavior change [26]. Lose it! (FitNow, Inc.) is a weight management app that will be used for weight self-monitoring [27], which has been used in weight loss programs in previous studies [20,28] and is available for free. WhatsApp, which is widely used in Côte d'Ivoire [29], can send and receive images, videos, audio, and text [30], and can deliver a variety of health education messages at low cost [31,32]. In addition, a step-counting app, such as Google Fit (Google Inc) for Android users and Health Care (Apple Inc) for iPhone users, can be installed for weight change and step-count monitoring. These apps will be installed on the smartphones of the intervention group, and participants will receive an orientation on their use. Additionally, they will be able to contact their supporters via WhatsApp to inquire about the use of Lose It! and another app at any time.

Health education should include recommendations for and emphasize the importance of a healthy diet, exercise, and weight measurement. Diet and exercise content should be in line with the “Diet and Exercise” described in the “Obesity” chapter of the Côte d'Ivoire National Disease-Specific Cookbook Guidelines [33]. In-person or telephonic health education will be conducted once a month and tailored to the stages of behavioral change [26] (precontemplation, contemplation, preparation, action, and maintenance) of the target population. Interventions corresponding to the change process [34-36] of each stage are provided. The intervention stimulates interest in weight loss during the precontemplation stage, motivates implementation during the contemplation stage, supports implementation during the preparation stage, supports continuation during the action stage, and supports maintenance during the maintenance stage to maintain confidence in behavior. The intervention and evaluation schedules are shown in Table 2.

To maintain consistency among supporters in the intervention, a manual was developed on how to send health education material via WhatsApp. A manual for telephone interviews was developed, and supporters will conduct telephone interviews according to the manual and provide a report on the content of the interviews. Supporters will be trained on the content of the intervention and the survey to ensure the quality of the intervention and the survey.

No interventions are administered in the control group. For the control group, height and weight are measured at baseline and at data collection at 3, 6, and 12 months, and BMI is calculated and reported on the spot by the supporter. This allows participants in the control group to know their weight and BMI regularly. In addition, after the 12-month survey is completed, scales will be installed in the target markets free of charge so that participants can continue to measure their weights. In addition, we will introduce the app to those who wish to use it.

**Table 1:** Adaptations of the weight loss program following the American Heart Association (AHA)/American College of Cardiology (ACC)/The Obesity Society (TOS) guidelines for the management of overweight and obesity in adults.

Variable	AHA/ACC/TOS guidelines [13]	Intervention adapted to this study	Reason for adaptation
Participants and setting	Individual with obesity who contacted with general practitioner or another medical organization	Community recruitment	Many individuals with obesity are unaware of their obesity risk and do not feel the need to lose weight, making them less likely to visit a health care facility voluntarily.
Intervention method and frequency	In-person, high-intensity (≥14 sessions in 6 months) sessions and counseling for feedback (face-to-face or telephonic)	6 phone calls and 3 face-to-face health education sessions (focused on behavior change stages, not full medical counseling)	For nonmedical interventions, feasibility was considered.
Intervention duration	6 months	6 months	No change
Interventionist	Health professionals (registered dietitians, psychologists, exercise specialists, health counselors, or professionals in training) who adhered to formal protocols in weight management	Trained non–medical professionals over 3 days, covering coaching, intervention methods by behavior change stage, and diet/exercise per Côte d'Ivoire obesity guidelines	Côte d'Ivoire’s limited medical personnel and resources were taken into consideration.
Weight loss goal	Loss of 5%-10% of baseline weight within 6 months	Loss of 2% of baseline weight within 6 months	It is a clinically meaningful minimum value and is feasible according to previous studies [8,19].
Physical activity	Advice by health professionals; increased aerobic physical activity (such as brisk walking) for ≥150 min	Recommend increasing the number of steps in one’s daily routine and establish a step count goal	With limited facilities and resources for fitness programs, the plan recommends daily walking and using a smartphone app for exercise.
Dietary intervention	Advice by health professionals; daily energy deficit of 500 kcal typically achieved with dietary intake of 1200-1500 kcal/d for women	Advice from trained non–health professionals based on the National Recipe Guidelines: offer recommended local dishes and cooking tips	The method is feasible for non–health professionals, using guidelines adapted to local eating habits without calorie calculations, which are difficult for participants and interventionists.

**Table 2:** Enrollment, intervention, and data collection timeline.

			–2 months		–1		0^a^		1		2		3		4		5		6		12
**Enrollment**																					
	Eligibility assessment	✓																			
	Informed consent	✓																			
	Allocation			✓																	
**Intervention**																					
	Distribution of scales					✓		✓													
	App installation and orientation					✓		✓													
	Health education via message app (1 time/wk)							✓		✓		✓		✓		✓		✓			
	In face-to-face health education (at the time of survey)					✓						✓						✓			
	Health education by phone							✓		✓				✓		✓					
**Data collection for intervention group**																					
	Collect data at time of call (weight, stage of behavior change)							✓		✓				✓		✓					
**Data collection for intervention group and control group**																					
	Weight, body fat percentage, and abdominal circumference measurements	✓				✓						✓						✓		✓	
	Height measurement	✓																			
	Structured interview	✓				✓						✓						✓		✓	

^a^The intervention lasted from months 0 to 6.

### Outcome

The primary end point of the study is to compare weight changes from baseline to the end of the intervention (6 months later) between the intervention and control groups.

Secondary end points include the differences in body fat percentage, abdominal circumference, stage of behavioral change in weight loss behaviors such as diet and exercise, percentage of individuals with correct weight perception, degree of body pain (with visual analog scale [VAS]), and self-rated health at the end of the intervention (6 months later) compared to baseline. Simultaneously, the intervention group will be evaluated in terms of the frequency of inputs to the self-monitoring apps, degree of participation in WhatsApp groups, average number of steps taken, and degree of weight loss during the intervention period.

To evaluate the interim process of the intervention, the intervention and control groups will be compared and analyzed in the same manner as described above, 3 months after the start of the intervention. In addition, the intervention group will be evaluated for weight change at 1, 2, 4, and 5 months and for changes in the behavioral change stage of weight loss behaviors such as diet and exercise.

As a follow-up evaluation after the intervention, both groups will be evaluated at 12 months (6 months after the end of the intervention) in the same way.

### Sample Size Calculation

For the sample size calculation, we relied on a meta-analysis [19] of a smartphone app–based weight loss intervention. We applied –1.5 (95% CI –2.09 to –1.09) kg as the clinically meaningful difference between the 6-month smartphone-only intervention without face-to-face motivational interviewing and the control group, as indicated in the meta-analysis. However, the meta-analysis did not provide SD values, so we used the SD (–1.8, SD 3.7) [37] value of the smartphone self-monitoring–only intervention results as a reference and set the expected value at –1.5 (SD 3.7) kg. Furthermore, the α error was set at 5% (95% CI=1.96), power (1 – β error) at 80% (0.842), and the dropout rate at 10%. Based on the above values and after consulting a statistical expert, the sample size was calculated to be 108 individuals for each group (N=216 in total).

### Assignment, Allocation Concealment, and Blinding

The random assignment will be performed using the permuted block method. Stratification will be conducted based on BMI (threshold: 32 kg/m^2^) and age (threshold: 45 years), which represent the median values reported in a previous study. The four resulting strata will be as follows: (1) BMI ≥32 and age ≥45 years, (2) BMI ≥32 and age <45 years, (3) BMI <32 and age ≥45 years, and (4) BMI <32 and age <45 years.

The list of potential participants recruited was sent to study member allocator 1 in Japan. Allocator 1 prepares a provisional ID list that does not contain attributes other than BMI and age to ensure concealment and sends it to allocator 2. Allocator 2 creates an allocation table with an allocation ratio of 1:1 and BMI and age as adjustment factors using software for randomized controlled trial–stratified substitution blocks and allocates the provisional IDs to each stratum in descending order of provisional ID numbers, from top to bottom.

This study will be conducted in an unblinded manner. This is because the intervention group will be given a scale and an app to be installed; therefore, blinding of the participants will not be possible. In addition, the content of the questions asked during each evaluation differs between the intervention and control groups, making blinding difficult for supporters and research team members.

### Data Collection Methods

Data will be obtained from supporters visiting the market to conduct face-to-face structured interviews (Multimedia Appendix 1) and anthropometric measurements. Body measurements will be taken at the time of recruitment to measure height and weight, and BMI will be calculated immediately. Subsequent data collection will include weight measurements, body fat percentage, and abdominal circumference. Height will be measured using a hardware SHUREMAN Lock Convex measuring tape (Kenoh Co, Ltd, Japan, model LC-5525) with a locking function, and an InBody (InBody Japan Inc, model H20N) will be used as the body composition scale. Structured interviews will be conducted using a questionnaire asking about sociodemographics, weight perception, behavioral change stages related to weight loss, behaviors related to weight management, body pain level (using the VAS scale), and the use of smartphone apps for weight management.

In addition, the intervention group will be asked about the weight and behavioral change stages of exercise and diet during the monthly telephone intervention.

### Statistical Methods

The primary outcome will be evaluated by comparing the difference in weight change between the intervention and control groups 6 months after the intervention ended. In addition, the effectiveness of the weight loss intervention will be estimated using a linear regression model with weight change at 6 months as the dependent variable and the affiliation group, stratification variables, and other sociodemographic attributes as covariates.

Among the secondary outcomes, weight, body fat percentage, abdominal circumference, and degree of body pain (VAS scale) will be measured and compared in the same way as the primary outcome at all evaluation periods (3, 6, and 12 months). Other secondary outcomes will be compared to differences in rates at baseline and 3, 6, and 12 months. These analyses will follow the intention-to-treat principles. Additionally, intention-to-treat and per-protocol analyses will be performed and compared to address cases of protocol noncompliance.

Within the intervention groups, weight loss will be used as the dependent variable, and linear regression model analyses will be performed to estimate the impact of self-monitoring input frequency, WhatsApp group participation, and sociodemographic characteristics as covariates.

### Data Management and Monitoring

All data will be collected on paper, entered into an Excel data entry format, and stored as digital data. The reason for this is that the intervention in this study involved self-monitoring of weight by app and encouraging behavioral change, so no significant safety issues are foreseen [38,39].

The research team will create a participant management sheet and continuously monitor the participants’ health and weight loss status during the supporters’ calls to identify any problems. During the study period, supporters will hold web-based meetings with the research team 1-6 times a month to share the participants’ situation, which will facilitate the detection of unexpected adverse events.

### Ethical Considerations

This protocol (version 6) and all related materials for this study were reviewed and approved by the Ethics Committee for Research on Life Sciences and Medicine Involving Human Subjects of Yokohama City University, Japan (approval General 2024-020) and by the National Ethics Committee in Health and Life Sciences of Côte d'Ivoire (RefNo 218-23/ MSHPCMU/CNESVS-km). In the event of a protocol change, the ethics committee and UMIN Clinical Trials Registry will be notified.

After receiving a full explanation face-to-face, the participant will be asked to sign an informed consent form. At the same time, the participant will be informed that she can withdraw her consent (Multimedia Appendix 2). The collected digital data will be password-protected, and the paper-based data will be stored in a locked cabinet. Additionally, the data for this research will only be accessible to the research team members who have been instructed to maintain strict confidentiality. The results of this research will be published in a peer-reviewed international scientific journal.

The study was registered in the UMIN Clinical Trials Registry (ID 000055142) (Multimedia Appendix 3).

## Results

In accordance with this protocol, the recruitment of participants started on August 26, 2024; by September 8, 2024, a total of 231 people were recruited. After reconfirming eligibility, one participant was excluded, and from 230 people, 108 were allocated to the intervention group and 108 were allocated to the control group. The baseline survey began on November 15, 2024, and is currently ongoing as of the end of November 2024.

## Discussion

### Expected Findings

The expected outcomes will analyze body weight, body fat percentage, and abdominal circumference. It is anticipated that the intervention group will achieve greater reductions in these three indicators compared to the control group. Additionally, the difference between the intervention and control groups is expected to be greater at 6 months and remain stable at 12 months.

### Comparisons With Prior Work

There are fewer community-based weight loss programs in sub-Saharan Africa than those conducted within health care facilities. One such program is that of Herrmann et al [25]; however, it primarily involves interventions led by health care professionals with specialized knowledge and does not use smartphones or web-based media.

In our study, we deliberately chose to use nonmedical staff as direct interventionists to evaluate the feasibility of implementing a weight loss program by those staff in sub-Saharan African countries where human resources in health care are particularly scarce.

Additionally, weight loss programs that use smartphone apps often recruit participants via web-based media, thereby primarily attracting individuals who are already motivated to lose weight [20]. Our study differs from previous research in that it targets communities with cultural norms that view being plump as a virtue, includes individuals who may not be aware of the need to lose weight, and recruits participants through face-to-face interactions.

To our knowledge, this is the first study to implement a smartphone app–based weight loss program in sub-Saharan Africa that does not require direct intervention by health care professionals and targets specific communities.

### Study Significance and Feasibility

The findings of this study will provide information on the effectiveness and feasibility of a smartphone app–based weight loss program for communities in sub-Saharan Africa. Additionally, it will offer evidence on the extent to which interventions led by nonmedical individuals can be effective.

In Côte d'Ivoire and neighboring countries, the most common occupation among women is merchant [40-43]. If the feasibility of using smartphone apps within this community is demonstrated, it could pave the way for applying it to other health programs. Furthermore, if the effectiveness of this program is confirmed, it has the potential to serve as a low-cost, sustainable weight loss model at the policy level.

### Limitations

First, the intervention cannot be blinded to the participants because those in the intervention group will receive a weighing scale. It is also difficult to make the supporters blinded because the questionnaires are different between the two groups. Second, it is not possible to perfectly prevent the participants from exchanging information with each other. However, we will try to understand the status of communications between the two groups by including a related question in the questionnaire. Third, the target population of the study may not represent all female merchants in Côte d'Ivoire as we only included those who have smartphones. However, as the smartphone ownership rate among female merchants in the market in the previous study was 77% [8], we judged this to be feasible.

## Appendix

Multimedia Appendix 1 Survey form (baseline, 3, 6, and 12 months later).

Multimedia Appendix 2 Consent form and related documents.

Multimedia Appendix 3 Table of WHO Trial Registration Data Set.

## Abbreviations

ACC: American College of Cardiology
AHA: American Heart Association
CONSORT-EHEALTH: Consolidated Standards of Reporting Trials of Electronic and Mobile Health Applications and Online Telehealth
NCD: noncommunicable disease
SNS: social networking service
SPIRIT: Standard Protocol Items: Recommendations for Interventional Trials
TOS: The Obesity Society
VAS: visual analog scale

## Data Availability

The datasets generated or analyzed during this study will be available from the corresponding author upon reasonable request.
