# Supplementary material for: Evaluation of a Smartphone-Based Weight Loss Intervention with Telephone Support for Merchant Women With Obesity in Côte d'Ivoire: Protocol for a Randomized Controlled Trial
Source: JMIR Res Protoc. 2025 Mar 18;14:e69264. doi: 10.2196/69264 (PMC11962317; doi:10.2196/69264)
Supplement: Multimedia Appendix 1 [file resprot_v14i1e69264_app1.pdf]

Commencement l'intervention

Nom d'enquêter :

Date: ID:

Avant le commencer l'enquete	
Groupe	1. Groupe d'intervention<br>2. Groupe de contrôle

Mesure		
Taille<br>…………………m	Poids<br>…………………Kg	IMC (dois etre Plus de 30)
Tour de taille<br>……………………cm	Taux de pourcentage du gras du corps<br>…………………………..%	

Observation		
1.	Qu'est ce qu'elle vend?	1.Nourriture 2.Vêtements 3.Pagnes 4.Pour la cusine<br>5.Pour le ménage 6. Coiffure 7.Stockage<br>8. Autres (précisez)

Questions sur la Sociodémographique		
2.	Date de naissances	
3.	Groupe ethnique (du répondant)	1. Akan 5. Gour (Voltaïque)<br>2. Krou 6. Africain de l'ouest<br>3. Mandé nord 7. Autre africain<br>4. Mandé sud 8. Non africain
4.	Niveau d'instruction (du répondant)	1. Cours primaire inachevé 6. Superieure achevée<br>2.Primaire achevé 7.Arabisant (cours coranique)<br>3.Secondaire inachevé 8. Sans éducation formelle<br>4. Secondaire achevé 9. Ne sait pas<br>5.Superieur inachevé 10.Autres (précisez)
5.	Statut matrimonial	1. Marié(e) 2. Divorcé(e) 3. Célibataire<br>4. Veuve/veuf 5. Autres
6.	Combien des personnes avez-vous dans votre foyer?	
7.	Je vous lis quelques intervalles de montants derevenus de ménage ; veuillez indiquer l'intervalle qui correspond à votre revenu (mensuel)?<br>[Lire les différentes options et entourer la réponse]	1. 35.000 6. 200.000 à 250.000<br>2. 35.000 à 50.000 7.250.000 à 300.000<br>3. 50.000 à 75.000 8. 300.000 à 400.000<br>4. 75.000 à 100.000 9. 400.000 à 500.000<br>5. 100.000 à 200.000 10. 500.000
8.	Le revenu familial mensuel des derniers mois. Choisissez parmi les options numérotées ci-dessus.	
9.	Combien de fois avez-vous accouché ?	……………… fois

Questions sur la conscience		
10.	Comment est votre état de santé en général d'aujourd'hui ?	1. Très bon 2. Bon 3. Moyen<br>4. Mauvais 5 Très mauvais

Commencement
l'intervention

11.	Comment pensez-vous votre poids (du corps) ?	1. maigre ou mince 3.Surpoids<br>2. Poids idéal 4. Très surpods (Obésité)
12.	Quel est votre poids idéal ?	(Après l'avoir informée de son poids actuel)<br>____________________Kg
13.	Combien de fois avez-vous manger hier (ou dernier jour de travaille) ?	……………………fois/jour
14.	Avez-vous consommé ces 12 derniers mois une boisson alcoolisée (bière, vin, liqueur, tchapalo, bandji etc.) ?	1. Oui<br>2. Non
15.	Si oui, durant les 12 derniers mois, combien de fois avez-vous bu ?	__________fois / année
16.	Combien de fois vous vous êtes pesé(e) par année ?	…………………fois /année
17.	Avez-vous le pèse-personne chez vous ?	1.Oui 2. Non 3.Ne sais pas
18.	Où êtes-vous pesé(e) pour la dernière fois ?	1. Chez-moi<br>2. A la pharmacie<br>3. A l'hopital ou la clinique<br>4. A la place de campagne/manifestation<br>5. Au marché ou sur la route (personne tourné avec la pèse)<br>6. Chez quelqu'un<br>7. Autres………………………………………………………………………
19.	Avez-vous reçu quel qu'information pour diminuer de poids ?	1.Oui 2. Non 3.Ne sais pas
20.	Si oui, par quelles occasions avez-vous reçu ?<br>(Plusieurs réponses seront possibles)	1. Par la television<br>2. Par le radio<br>3. Par le panonceau<br>4. Par le campaine de Diabete ou d'autre campaine<br>5. Par l'hopital<br>6. Par la pharmacie<br>7. Par l'eglise ou mosque<br>8. Autre______________________________________

Commencement
l’intervention

**Questions sur la maladie**		
21.	Avez-vous une ou des maladies en cours de traitement ?<br>（Plusieurs réponses possibles）	**1.** Non<br>**2.** Diabète<br>**3.** Augmentation de taux de sucre<br>**4.** Hypertension<br>**5.** Monter la tension<br>**6.** Hyperlipidémie<br>**7.** Cardiopathie<br>**8.** Maladie de la reine<br>**9.** Cancer<br>**10.** Autres……………………………………………………………
22.	Avez-vous mal aux hanches ?	1.Oui 2. Non 3.Ne sais pas
23.	Si oui, quelle est l'ampleur de votre douleur ?	Choisir entre 0 au 10 :<br>0, 1, 2, 3, 4, 5, 6, 7, 8, 9, 10
24.	Avez-vous mal aux dos ?	1.Oui 2. Non 3.Ne sais pas
25.	Si oui, quelle est l'ampleur de votre douleur ?	Choisir entre 0 au 10 :<br>0, 1, 2, 3, 4, 5, 6, 7, 8, 9, 10
26.	Avez-vous mal aux genoux ?	1.Oui 2. Non 3.Ne sais pas
27.	Si oui, quelle est l'ampleur de votre douleur ?	Choisir entre 0 au 10 :<br>0, 1, 2, 3, 4, 5, 6, 7, 8, 9, 10
**Questions Manegement pour le poids /Perception sur le poids**		
28.	Faites-vous de l'exercice ou avez-vous l’intention de faire exercice pour perdre du poids ou rester en forme ?	1. Je n'ai pas l'intention de le faire.<br>2. Je commencerai dans les 6 mois.<br>3. Je commencerai dans un mois<br>4. Je fais de l'exercice depuis moins de 6 mois<br>5. Je fais de l'exercice depuis plus de 6 mois
29.	Surveillez-vous votre alimentation ou avez-vous l’intention de surveiller votre alimentation pour perdre du poids et rester en bonne santé ?	1. Je n'ai pas l'intention de le faire.<br>2. Je commencerai dans les 6 mois.<br>3. Je commencerai dans un mois<br>4. Je surveille mon alimentation depuis moins de 6 mois<br>5. Je surveille mon alimentation depuis plus de 6 mois.
30.	Votre famille comprend-elle vos efforts pour perdre du poids ?	1. Oui, ils sont très compréhensifs.<br>2. Oui, un peu.<br>3. Non, pas très compréhensif.<br>4. Non, pas du tout.<br>5. Je ne sais pas.
**Question sur les application**		
31.	Avez-vous d’application pour le suivi du poids ?	1.Oui 2. Non
32.	Si oui, combiend fois par mois utilisez vous ?	______________Fois par mois
33.	Quel est le nom des application ?	*mettre tous les nom des applications<br>________________________

Apres 3mois

Date :
ID

Questions		Materiél	
Group		1. Groupe d'intervention<br>2. Groupe de contrôle	
**La question sur la conscience**			
**1.**	Comment est votre état de santé en général d'aujourd'hui ?	**1.** Très bon **2.** Bon **3.** Moyen<br>**4.** Mauvais **5.** Très mauvais	
**2.**	Comment pensez-vous votre poids (du corps)?	**1**. Maigre ou mince **3**.Surpoids<br>**2**. Poids idéal **4**. Tres surpods (Obésité)	
**3.**	Quel est votre poids idéal ?<br>(Après l'avoir informée de son poids actuel)	____________________Kg	
**4.**	Combien de fois avez-vous manger hier (ou dernier jour de travaille) ?	……………………………………………Fois	
**5.**	Avez-vous consommé ces 3 derniers mois une boisson alcoolisée ? (comme de la bière, du vin, de la liqueur, du cidre ou du koutoukou, du tchapalo, du bandji blanc ou du kendjous)	1. Oui<br>2. Non	
**6.**	Durant les 3 derniers mois, à quelle fréquence avez-vous bu au moins une boisson alcoolisée?<br>(LIRE LES REPONSES)	1. 5 jours ou plus/semaine<br>2. 1 à 4 jours / semaine<br>3. 1 à 3 jours /mois<br>4. Moins d'1 fois / mois	
**7.**	Avez-vous reçu des informations pour diminuer de poids ?	**1**.Oui **2**. Non **3**.Ne sais pas	
**8.**	Si oui, par quelles occasions avez-vous reçu ?<br>(Plusieurs réponses seront possibles)	1. Par la television<br>2. Par le radio<br>3. Par le panonceau<br>4. Par le campaine de Diabete ou d'autre campaine<br>5. Par l'hopital<br>6. Par la pharmacie<br>7. Par l'eglise ou mosque<br>8. Par les enquêteurs de cette recherche<br>9. Par les amis qui est participer cette recherche<br>10. Autre<br>______________________________	

Apres 3mois

Les questions sur la maladie			
9.	Avez-vous une ou des maladies en cours de traitement ? （Plusieurs réponses possibles）	**1.** Non<br>**2.** Diabète<br>**3.** Augmentation de taux de sucre<br>**4.** Hypertension<br>**5.** Monter la tension<br>**6.** Hyperlipidémie<br>**7.** Cardiopathie<br>**8.** Maladie de la reine<br>**9.** Cancer<br>**10.** Autres<br>……………………………………………………………………	
10.	Avez-vous mal aux hanches?	1.Oui 2. Non 3.Ne sais pas	
11.	Si oui, quelle est l'ampleur de votre douleur ?	Choisir entre 0 au 10 :<br>0, 1, 2, 3, 4, 5, 6, 7, 8, 9, 10	
12.	Avez-vous mal aux dos ?	1.Oui 2. Non 3.Ne sais pas	
13.	Si oui, quelle est l'ampleur de votre douleur ?	Choisir entre 0 au 10 :<br>0, 1, 2, 3, 4, 5, 6, 7, 8, 9, 10	
14.	Avez-vous mal aux genoux ?	1.Oui 2. Non 3.Ne sais pas	
15.	Si oui, quelle est l'ampleur de votre douleur ?	Choisir entre 0 au 10 :<br>0, 1, 2, 3, 4, 5, 6, 7, 8, 9, 10	
Questions Manegement pour le poids /Perception sur le poids			
16.	Faites-vous de l'exercice ou avez-vous l’intention de faire exercice pour perdre du poids ou rester en forme ?	1, je n'ai pas l'intention de le faire.<br>2, Je commencerai dans les 6 mois.<br>3, Je commencerai dans un mois<br>4, Je fais de l'exercice depuis moins de 6 mois<br>5, Je fais de l'exercice depuis plus de 6 mois	
17.	Surveillez-vous votre alimentation ou avez-vous l’intention de surveiller votre alimentation pour perdre du poids et rester en bonne santé ?	1, je n'ai pas l'intention de le faire.<br>2, Je commencerai dans les 6 mois.<br>3, Je commencerai dans un mois<br>4, Je surveille mon alimentation depuis moins de 6 mois<br>5, Je surveille mon alimentation depuis plus de 6 mois.	
18.	Votre famille comprend-elle vos efforts pour perdre du poids ?	1. Oui, ils sont très compréhensifs.<br>2. Oui, un peu.<br>3. Non, pas très compréhensif.<br>4. Non, pas du tout.<br>5. Non, je ne sais pas.	

Mesurément			
19.	Poids<br>………………………………Kg	Taux de % de graisse du cours<br>……………………………………%	Tours du taille<br>…………………………………cm

Apres 3mois

Question pour le **groupe d'intervention**		
**20.**	La situation du smartphone.<br>(S'il y a des problèmes du téléphone 3 dernier mois)	
**21.**	Quelle est la première date à laquelle vous avez saisi votre poids dans **"Lose it !"** ?<br>(Dehors du moment d'enquête de dernière foi.)	*Vérifiez-vous dans Lose it ! de son smartphone.<br>D/M/Y
**22.**	Quelle est la dernière date à laquelle vous avez saisi votre poids dans **"Lose it !"** ?	*Vérifiez-vous dans Lose it ! de son smartphone.<br>D/M/Y
**23.**	Combien de fois avez-vous enregistrez votre poids dans l'application de "**Lose it !"** pendant le 3 derniers mois ?<br>(Dehors du moment d'enquête de dernière fois	*Comptez dans Lose it ! de son smartphone<br><br>............................Fois/3 mois
**24.**	Combien de fois êtes-vous pesé pendant les 3 derniers mois ?<br>*Si elle a pesé plus que ce qui est enregistré sur Lose it !, posez la question.<br>(Dehors du moment d'enquête de dernière fois.)	............................Fois/3 mois
**25.**	Combien de fois avez-vous envoyé les **messages ou reactions** sur le group **"WhatsApp"** de recherche ?	*Comptez dans WhatsApp de son smartphone<br><br>............................Fois/3 mois
**26.**	Si avez-vous échange avec membre de recherche en prive, combien de fois avez-vous envoyé les messages.	*Comptez sur l'application de WhatsApp.<br><br>............................Fois/3 mois
**27.**	Combien de fois avez-vous recu d'appel par le membre d'equipe de recherche ?	*Vérifier avec enquêter.<br><br>............................Fois/3 mois
**28.**	Voir le **"Google fit"**ou **"Health care (Soin de santé)"**.<br>Notez le total pas de chaque mois.<br>(Si nous somme aujourd'hui le 15 Décembre, mentionnez jusuqu'a Décembre. Même si ce n'est pas à la fin de mois.)	2024<br>Novembre.................................pas<br>Décembre ....pas<br>2025<br>Janvier..pas<br>Février.......................................pas<br>Mars..pas

Apres 3mois

Question pour le **group contrôle**		
29.	Avez-vous le pèse-personne chez vous?	**1**.Oui **2**. Non **3**.Ne sais pas
30.	Combien de fois êtes-vous pesé au cours de ces 3 derniers mois ?<br>(Dehors du moment d'enquête de dernière fois.)	................................Fois/ 3 mois
31.	Où êtes-vous pesé(e) pour la dernière fois ?	**1.** Chez-moi<br>**2.** A la pharmacie<br>**3.** A l'hopital ou la clinique<br>**4.** A la place de campagne/manifestation<br>**5.** Au marché ou sur la route (personne tourné avec la pèse)<br>**6.** Chez quelqu'un<br>**7.** Avec les membres de la recherche<br>**8.** Autres<br>..
32.	Avez-vous d'application pour le suivi du poids ?	**1**.Oui **2**. Non
33.	Si oui, quel est le nom des application ?	*mettre tous les noms des applications<br>____
34.	Si oui, Qu'est-ce qui vous a insisté à installer cette application ?	1, Trouve moi-même<br>2, Appris par la famille ou des amis en dehors de ce marché<br>3, Appris par une amis dans ce marché qui est dans le groupe d'intervention.<br>4. Appris par une amis dans ce marché qui n'est dans le groupe d'intervention.<br>5,Autres (presiser)<br>____
35.	Si oui, combien de fois avez enregistrez votre poids dans cette application pendans 3 dernier mois ?	*Compter dans l'application<br>_____________Fois / 3 mois

Apres 6mois

Questions		Materiél	
Group		1. Groupe d'intervention<br>2. Groupe de contrôle	
La question sur la conscience			
1.	Comment est votre état de santé en général d'aujourd'hui ?	**1.** Très bon **2.** Bon **3.** Moyen<br>**4.** Mauvais **5.** Très mauvais	
2.	Comment pensez-vous votre poids (du corps)?	**1**. Maigre ou mince **3**.Surpoids<br>**2**. Poids idéal **4**. Tres surpods (Obésité)	
3.	Quel est votre poids idéal ?<br>(Après l'avoir informée de son poids actuel)	____________________________Kg	
4.	Combien de fois avez-vous manger hier (ou dernier jour de travaille) ?	....Fois	
5.	Avez-vous consommé ces 3 derniers mois une boisson alcoolisée ? (comme de la bière, du vin, de la liqueur, du cidre ou du koutoukou, du tchapalo, du bandji blanc ou du kendjous)	1. Oui<br>2. Non	
6.	Durant les 3 derniers mois, à quelle fréquence avez-vous bu au moins une boisson alcoolisée?<br>(LIRE LES REPONSES)	1. 5 jours ou plus/semaine<br>2. 1 à 4 jours / semaine<br>3. 1 à 3 jours /mois<br>4. Moins d'1 fois / mois	
7.	Avez-vous reçu des informations pour diminuer de poids ?	**1**.Oui **2.** Non **3**.Ne sais pas	
8.	Si oui, par quelles occasions avez-vous reçu ?<br>(Plusieurs réponses seront possibles)	1. Par la television<br>2. Par le radio<br>3. Par le panonceau<br>4. Par le campaine de Diabete ou d'autre campaine<br>5. Par l'hopital<br>6. Par la pharmacie<br>7. Par l'eglise ou mosque<br>8. Par les enquêteurs de cette recherche<br>9. Par les amis qui est participer cette recherche<br>10. Autre<br>____	

Apres 6mois

Les questions sur la maladie			
9.	Avez-vous une ou des maladies en cours de traitement ?　(Plusieurs réponses possibles)	**1.** Non<br>**2.** Diabète<br>**3.** Augmentation de taux de sucre<br>**4.** Hypertension<br>**5.** Monter la tension<br>**6.** Hyperlipidémie<br>**7.** Cardiopathie<br>**8.** Maladie de la reine<br>**9.** Cancer<br>**10.** Autres<br>………………………………………………………………	
10.	Avez-vous mal aux hanches?	1.Oui 2. Non 3.Ne sais pas	
11.	Si oui, quelle est l'ampleur de votre douleur ?	Choisir entre 0 au 10 :<br>0, 1, 2, 3, 4, 5, 6, 7, 8, 9, 10	
12.	Avez-vous mal aux dos ?	1.Oui 2. Non 3.Ne sais pas	
13.	Si oui, quelle est l'ampleur de votre douleur ?	Choisir entre 0 au 10 :<br>0, 1, 2, 3, 4, 5, 6, 7, 8, 9, 10	
14.	Avez-vous mal aux genoux ?	1.Oui 2. Non 3.Ne sais pas	
15.	Si oui, quelle est l'ampleur de votre douleur ?	Choisir entre 0 au 10 :<br>0, 1, 2, 3, 4, 5, 6, 7, 8, 9, 10	
Questions Manegement pour le poids /Perception sur le poids			
16.	Faites-vous de l'exercice ou avez-vous l'intention de faire exercice pour perdre du poids ou rester en forme ?	1, je n'ai pas l'intention de le faire.<br>2, Je commencerai dans les 6 mois.<br>3, Je commencerai dans un mois<br>4, Je fais de l'exercice depuis moins de 6 mois<br>5, Je fais de l'exercice depuis plus de 6 mois	
17.	Surveillez-vous votre alimentation ou avez-vous l'intention de surveiller votre alimentation pour perdre du poids et rester en bonne santé ?	1, je n'ai pas l'intention de le faire.<br>2, Je commencerai dans les 6 mois.<br>3, Je commencerai dans un mois<br>4, Je surveille mon alimentation depuis moins de 6 mois<br>5, Je surveille mon alimentation depuis plus de 6 mois.	
18.	Votre famille comprend-elle vos efforts pour perdre du poids ?	1. Oui, ils sont très compréhensifs.<br>2. Oui, un peu.<br>3. Non, pas très compréhensif.<br>4. Non, pas du tout.<br>5. Non, je ne sais pas.	
Mesurément			

19.	Poids<br>………………………………Kg	Taux de % de graisse du cours<br>……………………………………%	Tours du taille<br>…………………………………cm

Apres 6mois

Question pour le **groupe d'intervention**		
**20.**	La situation du smartphone.<br>(S'il y a des problèmes du téléphone 3 dernier mois)	
**21.**	Combien de fois avez-vous enregistrez votre poids dans l'application de "**Lose it !**" pendant le 3 derniers mois ?<br>(Dehors du moment d'enquête de dernière fois	*Comptez dans Lose it ! de son smartphone<br><br>..............................Fois/3 mois
**22.**	Combien de fois êtes-vous pesé pendant les 3 derniers mois ?<br>*Si elle a pesé plus que ce qui est enregistré sur Lose it !, posez la question.<br>(Dehors du moment d'enquête de dernière fois.)	..............................Fois/3 mois
**23.**	Combien de fois avez-vous envoyé les **messages ou reactions** sur le group **"WhatsApp"** de recherche ?	*Comptez dans WhatsApp de son smartphone<br><br>..............................Fois/3 mois
**24.**	Si avez-vous échange avec membre de recherche en prive, combien de fois avez-vous envoyé les messages.	*Comptez sur l'application de WhatsApp.<br><br>..............................Fois/3 mois
**25.**	Combien de fois avez-vous recu d'appel par le membre d'equipe de recherche ?	*Vérifier avec enquêter.<br><br>..............................Fois/3 mois
**26.**	Voir le **"Google fit"**ou **"Health care (Soin de santé)"**.<br>Notez le total pas de chaque mois.<br>(Si nous somme aujourd'hui le 15 Décembre, mentionnez jusuqu'a Décembre. Même si ce n'est pas à la fin de mois.)	2025<br>Janvier..pas<br>Février..pas<br>Mars...pas<br>Avril...pas<br>May...pas

Apres 6mois

Question pour le **group contrôle**		
27.	Avez-vous le pèse-personne chez vous?	**1.**Oui **2.** Non **3.**Ne sais pas
28.	Combien de fois êtes-vous pesé au cours de ces 3 derniers mois ?<br>(Dehors du moment d'enquête de dernière fois.)	……………………………Fois/ 3 mois
29.	Où êtes-vous pesé(e) pour la dernière fois ?	**1.** Chez-moi<br>**2.** A la pharmacie<br>**3.** A l'hopital ou la clinique<br>**4.** A la place de campagne/manifestation<br>**5.** Au marché ou sur la route (personne tourné avec la pèse)<br>**6.** Chez quelqu'un<br>**7.** Avec les membres de la recherche<br>**8.** Autres<br>…………………………………………………………………………
30.	Avez-vous d'application pour le suivi du poids ?	**1.**Oui **2.** Non
31.	Si oui, quel est le nom des application ?	*mettre tous les noms des applications<br>____
32.	Si oui, Qu'est-ce qui vous a insisté à installer cette application ?	1, Trouve moi-même<br>2, Appris par la famille ou des amis en dehors de ce marché<br>3, Appris par une amis dans ce marché qui est dans le groupe d'intervention.<br>4. Appris par une amis dans ce marché qui n'est dans le groupe d'intervention.<br>5,Autres (presiser)<br>_____________________________________
33.	Si oui, combien de fois avez enregistrez votre poids dans cette application pendans 3 dernier mois ?	*Compter dans l'application<br>____________Fois / 3 mois

Apres 12mois

Date
ID

Questions		Materiél	
Group		1. Groupe d'intervention<br>2. Groupe de contrôle	
**La question sur la conscience**			
**1.**	Comment est votre état de santé en général d'aujourd'hui ?	**1.** Très bon **2.** Bon **3.** Moyen<br>**4.** Mauvais **5.** Très mauvais	
**2.**	Comment pensez-vous votre poids (du corps)?	**1.** Maigre ou mince **3.**Surpoids<br>**2.** Poids idéal **4.** Tres surpods (Obésité)	
**3.**	Quel est votre poids idéal ?<br>(Après l'avoir informée de son poids actuel)	____________________________Kg	
**4.**	Combien de fois avez-vous manger hier (ou dernier jour de travaille) ?	....Fois	
**5.**	Avez-vous consommé ces 3 derniers mois une boisson alcoolisée ? (comme de la bière, du vin, de la liqueur, du cidre ou du koutoukou, du tchapalo, du bandji blanc ou du kendjous)	1. Oui<br>2. Non	
**6.**	Durant les 3 derniers mois, à quelle fréquence avez-vous bu au moins une boisson alcoolisée?<br>(LIRE LES REPONSES)	1. 5 jours ou plus/semaine<br>2. 1 à 4 jours / semaine<br>3. 1 à 3 jours /mois<br>4. Moins d'1 fois / mois	
**7.**	Avez-vous reçu des informations pour diminuer de poids ?	**1.**Oui **2.** Non **3.**Ne sais pas	
**8.**	Si oui, par quelles occasions avez-vous reçu ?<br>(Plusieurs réponses seront possibles)	1. Par la television<br>2. Par le radio<br>3. Par le panonceau<br>4. Par le campaine de Diabete ou d'autre campaine<br>5. Par l'hopital<br>6. Par la pharmacie<br>7. Par l'eglise ou mosque<br>8. Par les enquêteurs de cette recherche<br>9. Par les amis qui est participer cette recherche<br>10. Autre<br>____	

Apres 12mois

Les questions sur la maladie			
9.	Avez-vous une ou des maladies en cours de traitement ? （Plusieurs réponses possibles）	**1.** Non<br>**2.** Diabète<br>**3.** Augmentation de taux de sucre<br>**4.** Hypertension<br>**5.** Monter la tension<br>**6.** Hyperlipidémie<br>**7.** Cardiopathie<br>**8.** Maladie de la reine<br>**9.** Cancer<br>**10.** Autres<br>……………………………………………………………………	
10.	Avez-vous mal aux hanches?	1.Oui 2. Non 3.Ne sais pas	
11.	Si oui, quelle est l'ampleur de votre douleur ?	Choisir entre 0 au 10 :<br>0, 1, 2, 3, 4, 5, 6, 7, 8, 9, 10	
12.	Avez-vous mal aux dos ?	1.Oui 2. Non 3.Ne sais pas	
13.	Si oui, quelle est l'ampleur de votre douleur ?	Choisir entre 0 au 10 :<br>0, 1, 2, 3, 4, 5, 6, 7, 8, 9, 10	
14.	Avez-vous mal aux genoux ?	1.Oui 2. Non 3.Ne sais pas	
15.	Si oui, quelle est l'ampleur de votre douleur ?	Choisir entre 0 au 10 :<br>0, 1, 2, 3, 4, 5, 6, 7, 8, 9, 10	
Questions Manegement pour le poids /Perception sur le poids			
16.	Faites-vous de l'exercice ou avez-vous l'intention de faire exercice pour perdre du poids ou rester en forme ?	1, je n'ai pas l'intention de le faire.<br>2, Je commencerai dans les 6 mois.<br>3, Je commencerai dans un mois<br>4, Je fais de l'exercice depuis moins de 6 mois<br>5, Je fais de l'exercice depuis plus de 6 mois	
17.	Surveillez-vous votre alimentation ou avez-vous l'intention de surveiller votre alimentation pour perdre du poids et rester en bonne santé ?	1, je n'ai pas l'intention de le faire.<br>2, Je commencerai dans les 6 mois.<br>3, Je commencerai dans un mois<br>4, Je surveille mon alimentation depuis moins de 6 mois<br>5, Je surveille mon alimentation depuis plus de 6 mois.	
18.	Votre famille comprend-elle vos efforts pour perdre du poids ?	1. Oui, ils sont très compréhensifs.<br>2. Oui, un peu.<br>3. Non, pas très compréhensif.<br>4. Non, pas du tout.<br>5. Non, je ne sais pas.	

Mesurément			
19.	Poids ………………………………Kg	Taux de % de graisse du cours ……………………………………%	Tours du taille …………………………………cm

Apres 12mois

Question pour le **groupe d'intervention**		
**20.**	La situation du smartphone.<br>(S'il y a des problèmes du téléphone 6 dernier mois)	
**21.**	Combien de fois avez-vous enregistrez votre poids dans l'application de "**Lose it !**" pendant le 6 derniers mois ?<br>(Dehors du moment d'enquête de dernière fois	*Comptez dans Lose it ! de son smartphone<br><br>..............................Fois/6 mois
**22.**	Combien de fois êtes-vous pesé pendant les 6 derniers mois ?<br>*Si elle a pesé plus que ce qui est enregistré sur Lose it !, posez la question.<br>(Dehors du moment d'enquête de dernière fois.)	..............................Fois/6 mois
**23.**	Combien de fois avez-vous envoyé les **messages ou reactions** sur le group **"WhatsApp"** de recherche ?	*Comptez dans WhatsApp de son smartphone<br><br>..............................Fois/6 mois
**24.**	Si avez-vous échange avec membre de recherche en prive, combien de fois avez-vous envoyé les messages.	*Comptez sur l'application de WhatsApp.<br><br>..............................Fois/6 mois
**25.**	Combien de fois avez-vous recu d'appel par le membre d'equipe de recherche ?	*Vérifier avec enquêter.<br><br>..............................Fois/6 mois
**26.**	Voir le **"Google fit"**ou **"Health care (Soin de santé)"**.<br>Notez le total pas de chaque mois.<br>(Si nous somme aujourd'hui le 15 Décembre, mentionnez jusuqu'a Décembre. Même si ce n'est pas à la fin de mois.)	2025<br>..pas<br>..pas<br>..pas<br>..pas<br>..pas<br>..pas

Apres 12mois

Question pour le **group contrôle**		
27.	Avez-vous le pèse-personne chez vous?	**1.**Oui **2.** Non **3.**Ne sais pas
28.	Combien de fois êtes-vous pesé au cours de ces 3 derniers mois ?<br>(Dehors du moment d'enquête de dernière fois.)	................................Fois/ 3 mois
29.	Où êtes-vous pesé(e) pour la dernière fois ?	**1.** Chez-moi<br>**2.** A la pharmacie<br>**3.** A l'hopital ou la clinique<br>**4.** A la place de campagne/manifestation<br>**5.** Au marché ou sur la route (personne tourné avec la pèse)<br>**6.** Chez quelqu'un<br>**7.** Avec les membres de la recherche<br>**8.** Autres<br>..
30.	Avez-vous d'application pour le suivi du poids ?	**1.**Oui **2.** Non
31.	Si oui, quel est le nom des application ?	*mettre tous les noms des applications<br><br>____
32.	Si oui, Qu'est-ce qui vous a insisté à installer cette application ?	1, Trouve moi-même<br>2, Appris par la famille ou des amis en dehors de ce marché<br>3, Appris par une amis dans ce marché qui est dans le groupe d'intervention.<br>4. Appris par une amis dans ce marché qui n'est dans le groupe d'intervention.<br>5,Autres (presiser)<br><br>____
33.	Si oui, combien de fois avez enregistrez votre poids dans cette application pendans 3 dernier mois ?	*Compter dans l'application<br><br>____________Fois / 6 mois
