# Supplementary material for: Evaluation of a Smartphone-Based Weight Loss Intervention with Telephone Support for Merchant Women With Obesity in Côte d'Ivoire: Protocol for a Randomized Controlled Trial
Source: JMIR Res Protoc. 2025 Mar 18;14:e69264. doi: 10.2196/69264 (PMC11962317; doi:10.2196/69264)
Supplement: Multimedia Appendix 2 [file resprot_v14i1e69264_app2.pdf]

Version 6：Le 24 juillet 2024

Fiche de Renseignement

Intitulé de l'Etude :

Efficacité d'un programme de perte de poids basé sur une application smartphone pour les femmes commerçantes obèses en Côte d'Ivoire - un essai contrôlé randomisé (ECR)

1. Autorisations d'étude

L'étude est autorisée par le chef de la Chefferie d'Anono et le chef de marché d'Anono. Et encore, cette étude a été approuvée par le Comité National d'Ethique de Sciences de la Vie et de la Santé (CNESVS).

2. Responsable d'étude

KANOYA Yuka, Ph.D., Professeur de Université de Cité de Yokohama au japon

3. L'objective et l'intérêt d'étude

Développer un programme de perte de poids basé sur l'utilisation d'application pour smartphone en Côte d'Ivoire et déterminer son efficacité. En ciblant les femmes commerçantes travaillant au marché, la faisabilité de la mise en œuvre de ce programme de santé basé sur l'utilisation d'application pour smartphone dans les groupes à faible ou moyen revenu sera également examinée.

4. La méthodologie et période

(1) Méthodologie

L'étude est une enquête menée auprès de commerçantes obèses âgées de 18 ans et plus sur le marché d'Anono afin de déterminer l'efficacité du programme de perte de poids. Parmi les personnes ayant données leur accord pour l'étude, celles qui sont éligibles seront divisées en deux groupes : celles qui participeront au programme de perte de poids et celles qui n'y participeront pas. Vous ne pouvez pas choisir votre groupe. Les personnes qui participeront au programme de perte de poids utiliseront une application smartphone pour le programme. Elles utiliseront également WhatsApp pour communiquer avec l'équipe de recherche en cas de besoin.

Les participantes au programme rempliront des questionnaires et prendront des mesures du poids, de la taille, du pourcentage de graisse corporelle et de tour de taille au début de l'étude et après 3, 6 et 12 mois. Le questionnaire portera sur les caractéristiques sociodémographiques, le poids et des questions relatives à la santé. Les lieux où se déroulent les questionnaires, les pesées, etc. sont adaptés aux souhaits du groupe cible. L'enquête durera environ 10 à 40 minutes.

(2) La période

L'étude débutera en aoûtp 2024. Le programme de perte de poids dure six mois. Des enquêtes de collecte de données sont menées au début de l'intervention, après 3, 6 et 12 mois.

5. La cible d'étude

Les femmes commerçantes du marché Anono âgées de 18 ans et plus et dont l'IMC est supérieur ou égal à 30. Les femmes enceintes et allaitantes ne sont pas éligibles.

6. Les avantages et les inconvénients individuels de cette étude

Si vous participez à cette étude, vous devrez consacrer un certain temps à remplir des questionnaires et

à prendre des mesures du poids, de la taille, du pourcentage de graisse corporelle et de tour de taille. C'est pourquoi nous vous informerons à l'avance de l'heure de l'entretien et nous nous efforcerons de la respecter. Si vous vous sentez dépassée par les questions posées lors de l'entretien, vous n'êtes pas obligées d'y répondre. En prenant part à l'étude, vous pourrez participer gratuitement à des mesures régulières de votre poids, ce qui vous aidera à gérer votre propre santé.

Les membres du groupe participant au programme de perte de poids utiliseront une application pour smartphone afin d'entrer des données dans l'application et de communiquer avec l'équipe de recherche via WhatsApp. En participant au programme de perte de poids, vous apprendrez et mettrez en pratique des connaissances et des méthodes liées à la santé. En outre, on peut s'attendre à ce que la perte de poids prévienne les maladies et conduise à une vie de plus en plus saine.

7. Retirer le consentement

Vous pouvez vous retirer à tout moment après avoir accepté de coopérer à cette étude, ou même pendant votre participation, jusqu'à la fin du questionnaire après 6 mois de programme de perte de poids. Si vous décidez de ne plus participer à l'étude, quelle qu'en soit la raison, vous pouvez vous retirer en utilisant le "formulaire de retrait de consentement" (ci-joint). Dans ce cas, nous détruirons immédiatement les données que vous nous avez fournies. Toutefois, si le questionnaire et les mesures physiques pour le sixième mois du programme de perte de poids ont été complétés au moment où vous demandez le retrait du consentement, les données ne peuvent pas être identifiées et ne peuvent pas être supprimées des données.

8. Aucun désavantage

Vous n'avez aucun désavantage et sanction, si vous ne participez pas à cette étude.

9. Confidentialité et traitements des données à caractère personnel

Dans le cadre de cette étude, nous vous demanderons les noms des personnes afin de pouvoir répéter le questionnaire auprès des mêmes sujets. Nous utiliserons également les réponses que vous fournirez dans le questionnaire aux fins de cette étude. Nous vous assurons également que votre nom et prénoms et d'autres informations ne seront pas divulgués et seront strictement protégés. Lorsqu'elles seront utilisées dans le cadre de la recherche, les informations seront traitées avec un numéro de recherche (numéro d'identification). Une table de correspondance sera établie entre le nom individuel et le numéro d'identification. Si cette table de correspondance et d'autres informations, etc., sont stockées sur des supports électroniques, elles le sont sur l'ordinateur du chercheur principal, qui est verrouillé et dont le fichier électronique est protégé par un mot de passe. La pièce où se trouve cet ordinateur a un accès contrôlé et n'est pas accessible à des tiers. Les informations obtenues dans le cadre de cette recherche seront gérées de la même manière que le tableau correspondant.

Les données et autres informations obtenues dans le cadre de cette recherche peuvent être partagées à l'avenir avec des institutions de recherche et des chercheurs impliqués dans la recherche et le développement dans le domaine de la santé et de la médecine, dans le pays et à l'étranger, et peuvent être utilisées à des fins secondaires dans un large éventail de recherche et de développement dans le domaine de la santé et de la médecine. Dans ce cas, un nouveau protocole de recherche sera préparé et la pertinence et l'éthique de la recherche seront approuvées par le comité d'évaluation éthique approprié en

fonction du contenu du nouveau plan de recherche. Nous veillerons également à ce que vous disposiez d'une procédure pour obtenir le consentement d'une manière appropriée approuvée par le comité d'évaluation éthique, ou que le plan de recherche soit mis à la disposition du public et que vous ayez la possibilité de refuser d'utiliser les informations dans le cadre de la recherche. Si vous changez d'avis après avoir donné votre consentement cette fois-ci, vous pouvez nous en informer à tout moment. Nous cesserons rapidement d'utiliser les données vous concernant. Cependant, si les données sont déjà utilisées pour la recherche et le développement au moment de ta demande, nous ne pourrons pas arrêter l'utilisation des données. Notez que les noms et autres informations ont été retirés des échantillons et des informations fournies, et qu'ils ne contiennent pas d'informations permettant d'identifier immédiatement des personnes.

En signant ce document de consentement, vous consentez également à l'utilisation secondaire.

10. La garde et l'annulation de données

Les informations seront conservées de manière strictement confidentielle jusqu'à la fin de la période de recherche. Les informations obtenues dans le cadre de cette étude ne seront pas personnellement identifiables et tous les fichiers de données électroniques et les outils de stockage des données, etc. seront protégés par un mot de passe. Les données seront également stockées et gérées dans un casier fermé à clé au domicile du chercheur principal.

Les informations seront conservées jusqu'à cinq ans après la date d'achèvement de l'étude. Les informations compilées pour l'analyse des données seront conservées pour une durée indéterminée après la période de stockage, en raison de la possibilité d'une utilisation secondaire. Les informations sur papier seront numérisées, déchiquetées et éliminées dès que possible.

11. Conflit d'intérêts

Le conflit d'intérêts est une situation dans laquelle un tiers peut craindre que la recherche ne soit pas menée de manière équitable et appropriée, comme la falsification de données de recherche ou le traitement préférentiel de certaines entreprises, en raison d'intérêts financiers avec des parties externes, etc.

Bien que cette recherche soit soutenue par une subvention pour la recherche scientifique (Grant-in-Aid for Scientific Research Fund) obtenue par le chercheur principal, les intérêts de la subvention pour la recherche scientifique ne sont pas prioritaires et l'équité de la recherche n'est pas remise en cause.

12. Consultation des participants à la recherche et d'autres personnes concernées

Si vous avez des questions ou des inquiétudes concernant cette recherche, n'hésitez pas à contacter le responsable d'étude. Toutefois, il se peut que nous ne soyons pas en mesure de vous donner une réponse en raison d'informations personnelles concernant d'autres chercheurs, etc. ou pour protéger les droits de propriété intellectuelle du chercheur. Dans ce cas, le chercheur principal fournira une explication.

13. Financement de l'étude

Cette étude est financée par la subvention d'aide pour la recherche scientifique par le gouvernement japonais. Toutefois il n'existe pas un conflit d'intérêts.

14. Publier le résultat des études

Les résultats de la recherche peuvent être présentés et publiés dans des réunions et des articles scientifiques afin de contribuer à l'amélioration de la santé des populations d'Afrique subsaharienne. Toutefois, nous garantissons que votre nom et vos autres informations personnelles ne seront pas rendus publics et seront strictement protégés.

15. Droit de la Propriété intellectuelle

Le droit de la propriété intellectuelle sera lié pour l'organisation de l'étude

Contact :

Contact concernant le contenu de la recherche.
USUI Rui Ph.D., Chercheur visitant de l'Université de SHONAN Médical Science au Japon
Téléphone : ＋225-0594281408 Email : usuir@yokohama-cu.ac.jp

Contact concernant l'évaluation éthique
Comité National d'Éthique des Sciences de la Vie et de la Santé (CNESVS), Côte d'ivoire
Téléphone : +225 69 28 57 53/41 40 05 55 Email : cnesvscotedivoire@gmail.com

Formulaire de Consentement Eclairé

Je soussigné, après avoir reçu les explications du contenu d’étude de « Efficacité d'un programme de perte de poids basé sur une application smartphone pour les femmes obèses en Côte d'Ivoire - un essai contrôlé randomisé. » sur la base de « Fiche de Renseignement » avec les points ci-dessous exprime mon accord pour participer et collaborer.

☐ 1 Autorisations d’étude
☐ 2 Responsable d’étude
☐ 3 L’objective et l’intérêt et l’étude
☐ 4 La méthodologie et période
☐ 5 Le cible d’étude
☐ 6 Les avantages et les inconvénients individuels de cette étude
☐ 7 Retirer le consentement
☐ 8 Aucun désavantage
☐ 9 Confidentialité et traitements des données à caractère personnel
☐10 Le garde et l’annulation de données
☐11 Conflit d'intérêts
☐12 Consultation des participants à la recherche
☐13 Financement de l’étude
☐14 Publier le résultat des études
☐15 Droit de la Propriété intellectuelle

Contenu de la participation et de la coopération : entretien mené par l’enquêteur et mesure de l’IMC (Indice de Masse Corporelle), du pourcentage de graisse corporelle et du tour de taille.

Date: / /

Signature du participant

Nom et signature d’explicateur

Pour plus d’information veuillez contacter ci-dessous. Vous pouvez poser toutes les questions que vous voulez concernant cette étude, et si vous avez d’autres questions n’hésitez pas à contacter ;

Responsable d’étude
KANOYA Yuka, Ph.D., Professeur de Université de Cites de Yokohama au japon
Email：ykano@yokohama-cu.ac.jp
Contact concernant le contenu de la recherche.
USUI Rui Ph.D., Chercheuse visitant Université de Cites de Yokohama au japon
Email : usuir@yokohama-cu.ac.jp

Version 6：Le 24 juillet 2024

Formulaire de Retrait de Consentement

La présidente de l'université de
Université de Cites de Yokohama au Japon.

Je retire par la présente mon consentement pour participer à l'étude ci-dessous.

Intitulé de l'Etude :
« Efficacité d'un programme de perte de poids basé sur une application smartphone pour les femmes obèses en Côte d'Ivoire - un essai contrôlé randomisé ».

Responsables d'étude :

KANOYA Yuka, Ph.D., Professeur de Université de Cites de Yokohama au japon

Je retire mon consentement concerne ;

☐ Le traitement de toutes les données personnelles.

Date: / /

Signature du participant

＊Si vous pensez que vous voulez arrêter la participation, veuillez soumettre cette formulaire ou le document qui présenter la même teneur aux enquêteur.
