# Supplementary material for: Evaluation of a Smartphone-Based Weight Loss Intervention with Telephone Support for Merchant Women With Obesity in Côte d'Ivoire: Protocol for a Randomized Controlled Trial
Source: JMIR Res Protoc. 2025 Mar 18;14:e69264. doi: 10.2196/69264 (PMC11962317; doi:10.2196/69264)
Supplement: Multimedia Appendix 3 [file resprot_v14i1e69264_app3.pdf]

WHO Trial Registration Data Set

1.	Primary registry and trail identifying number	UMIN Clinical Trials Registry (UMIN-CTR) ID : 000055142
2.	Date of registration in primary registry	01, December,2024
3.	Secondary identifying number	None
4.	Source of monetary or material support	Japan Society for the Promotion of Science KAKENHI (Grants-in-Aid for Scientific Research of Japan)
5.	Primary sponsor	Japan Society for the Promotion of Science KAKENHI (Grants-in-Aid for Scientific Research of Japan)
6.	Secondary sponsor	None
7.	Contact for public queries	ykano@yokohama-cu.ac.jp
8.	Contact for scientific queries	usuir@yokohama-cu.ac.jp
9.	Public title	A Smartphone-Based Weight Loss Program with Phone Support for Women with Obesity in Côte d'Ivoire
10.	Scientific title	Evaluation of a Smartphone-Based Weight Loss Intervention with Phone-Call Support for Merchant Women With Obesity in Côte d'Ivoire: A Randomized Controlled Trial
11.	Countries of recruitment	Côte d'Ivoire
12.	Health condition or problem studied	Obesity
13.	Intervention	Weight Loss intervention focused on promoting weight measurement, messages, and phone calls via smartphone application.<br>- Intervention group: 6-month Weekly messages and monthly phone calls<br>- Control group: No intervention
14.	Key inclusion and exclusion criteria	Inclusion criteria<br>- Age: 18-65 years.<br>- Sex: Women<br>- BMI ≥ 30<br>- Working in Market A<br>Exclusion criteria:<br>- Pregnant or lactating women<br>- Weight loss is contraindicated<br>- Using physician – prescribed weight loss medication.
15.	Study type	- Intervention Study<br>- Randomised controlled trial (RCT)<br>- Parallel assignments into intervention and control groups<br>- Behavioral intervention does not allow blinding
16.	Date of first enrolment	26. Augst 2024
17.	Target sample size	2 x 108 individuals
18.	Recruitment status	Completed on 8 September 2024
19.	Primary outcome	Weight changes
20.	Key secondary outcomes	Body fat percentage, abdominal circumference, stage of behavioral change in weight loss behaviors
21.	Ethics Review	- Ethics Committee for Research on Life Sciences and Medicine Involving Human Subjects of Yokohama City University, Japan<br>- National Ethics Committee in Health and Life Sciences of Côte d'Ivoire
22.	Completion date	End of December 2025
23.	Summary Results	Research is currently underway.

24.	IPD sharing statement	Plan to share IPD : No
